# Natural Compounds and PCL Nanofibers: A Novel Tool to Counteract Stem Cell Senescence

**DOI:** 10.3390/cells10061415

**Published:** 2021-06-07

**Authors:** Emanuela Bellu, Sara Cruciani, Giuseppe Garroni, Francesca Balzano, Rosanna Satta, Maria Antonia Montesu, Angela Fadda, Maurizio Mulas, Giorgia Sarais, Pasquale Bandiera, Carlo Ventura, Martin Kralovič, Jan Sabo, Evzen Amler, Margherita Maioli

**Affiliations:** 1 Department of Biomedical Sciences, University of Sassari, Viale San Pietro 43/B, 07100 Sassari, Italy; ema.bellu@hotmail.it (E.B.); sara.cruciani@outlook.com (S.C.); giugarroni21@gmail.com (G.G.); mariafrancesca22@virgilio.it (F.B.); bandiera@uniss.it (P.B.); 2 Department of Medical, Surgical and Experimental Sciences, University of Sassari, 07100 Sassari, Italy; rsatta@uniss.it (R.S.); mmontesu@uniss.it (M.A.M.); 3 Istituto di Scienze delle Produzioni Alimentari (ISPA), Consiglio Nazionale delle Ricerche (CNR), Traversa la Crucca 3, 07100 Sassari, Italy; angela.fadda@cnr.it; 4 Department of Agriculture, University of Sassari, Via De Nicola 9, 07100 Sassari, Italy; mmulas@uniss.it; 5 Department of Life and Environmental Sciences, University of Cagliari, University Campus, 09042 Monserrato (Cagliari), Italy; gsarais@unica.it; 6 Laboratory of Molecular Biology and Stem Cell Engineering-Eldor Lab, National Institute of Biostructures and Biosystems, Innovation Accelerator, CNR, Via Piero Gobetti 101, 40129 Bologna, Italy; ventura.vid@gmail.com; 7 Institute of Biophysics, 2nd Faculty of Medicine, Charles University, V Uvalu 84, 150 06 Prague 5, Czech Republic; mkralovic@centrum.cz; 8 UCEEB, Czech Technical University, Trinecka 1024, 273 43 Bustehrad, Czech Republic; 9 Department of Medical and Clinical Biophysics, Faculty of Medicine, Pavol Jozef Šafárik University, Trieda SNP 1, 04011 Košice, Slovakia; jan.sabo@upjs.sk; 10 Center for Developmental Biology and Reprogramming (CEDEBIOR), Department of Biomedical Sciences, University of Sassari, Viale San Pietro 43/B, 07100 Sassari, Italy

**Keywords:** stem cells, cell senescence, nanofibers, natural extracts, skin aging, cellular mechanisms

## Abstract

Tissue homeostasis mainly depends on the activity of stem cells to replace damaged elements and restore tissue functions. Within this context, mesenchymal stem cells and fibroblasts are essential for maintaining tissue homeostasis in skin, in particular in the dermis. Modifications in collagen fibers are able to affect stem cell features. Skin properties can be significantly reduced after injuries or with aging, and stem cell niches, mainly comprising extracellular matrix (ECM), may be compromised. To this end, specific molecules can be administrated to prevent the aging process induced by UV exposure in the attempt to maintain a youngness phenotype. NanoPCL-M is a novel nanodevice able to control delivery of Mediterranean plant myrtle (*Myrtus communis* L.) extracts. In particular, we previously described that myrtle extracts, rich in bioactive molecules and nutraceuticals, were able to counteract senescence in adipose derived stem cells. In this study, we analyzed the effect of NanoPCL-M on skin stem cells (SSCs) and dermal fibroblasts in a dynamic cell culture model in order to prevent the effects of UV-induced senescence on proliferation and collagen depot. The BrdU assay results highlight the significantly positive effect of NanoPCL-M on the proliferation of both fibroblasts and SSCs. Our results demonstrate that-M is able to preserve SSCs features and collagen depot after UV-induced senescence, suggesting their capability to retain a young phenotype.

## 1. Introduction

Aging is associated with the progressive loss of function and higher stress sensitivity of the involved tissues. It covers many cellular types and signaling pathways affecting phenotypic changes, and ultimately human life [1]. Human skin, a complex tissue comprising dermis and epidermis, represents the tissue most exposed to environmental damage, such as UV light. The epidermis, the upper layer of the skin, attenuates the impact of molecular damage accumulation by rapid keratinocyte turnover. Conversely, the dermis accumulates damage, modifying tissue elasticity and stiffness, and cell features [2].

The aging process arising from environmental influences is called an extrinsic process and can be related to mechanisms of a stress response associated with lifestyle and nutrition [3]. One of the main exogenous stimuli is represented by sunlight exposure, since UV radiation produces photo-oxidative stress mechanisms on skin cell populations, deeply modifying extracellular matrix features [4]. Stem cells play a central role in preserving tissue homeostasis due to their ability to replace damaged elements and restore tissue function. These properties can be significantly reduced after UV exposure and oxidative-stress-related dysfunctions [5]. Stem cells are able to undergo self-renewal or commit toward a specific differentiated phenotype upon physical or chemical stimuli [6,7,8,9,10], exhibiting different plasticity and differentiation potential, according to their tissue source [6,10,11,12,13]. Skin comprises several stem cell types, showing different roles, properties, and differentiation capabilities [14,15]. In particular, skin stem cells (SSCs), a mesenchymal cell population, are able to undergo different fates due to their asymmetric division: one cell can maintain the undifferentiated condition in the niche, while the other can be recruited into becoming a differentiated cell [12]. SSCs were found both in the epidermis and dermis. However, in the epidermis, the majority of stem cells have an epithelial origin, and a fluctuating amount of mesenchymal stem cells can be found in the epidermis in undamaged skin, increasing during inflammation, or wound processes [16]. After stress and related inflammatory responses, stem cells of epithelial origin undergo epithelial–mesenchymal transition. They express mesenchymal markers and features, and migrate from the niches to the damaged area, thus contributing to restoring tissue homeostasis [16]. UV exposure is able to deeply modify the molecular behavior of stem cells and the niche properties [17].

Extracellular matrix (ECM) changes actively contribute to SSC behavior. Interestingly, stem cell niches mainly comprise the ECM, which is involved in maintaining a complex network of molecules able to regulate stem cell proliferation and differentiation [18]. The ECM is responsible for the structural and mechanical properties of the entire tissue, and is primarily produced by dermal fibroblasts, which, during extrinsic aging, experience cellular damage, affording changes in ECM composition and remodeling [19,20]. Nevertheless, in addition to changes in collagen fibril organization [21], the dermis gradually becomes atrophic, decreasing in thickness and in hyaluronic acid production [22].

Along with hyaluronic acid, changes in collagen fibers modify the niche environment [18]. The most abundant types of collagen are Collagen I and III, whereas the basal membrane contains mainly collagen IV, able to optimize cell polarization and functions. Within this context, a lower capability of pro-collagen I production [23], together with age-specific changes in collagen turnover [24], have been previously described in aged cultured fibroblasts.

Within this context, solar damage is associated with a reduced hyaluronic acid synthesis owing to the downregulation of the Hyaluronan Synthase 2 (HAS2) gene. The HAS2 gene is expressed both in fibroblasts and SSCs, encoding the most relevant enzyme in hyaluronan production. Moreover, its expression is important for cell cycle progression, and G1 phase, counteracting cellular senescence, preserving cell viability, and regulating apoptosis under stress stimuli [25]. In addition to HAS2, other genes are involved in skin protection from photoaging, including, for example, those encoding sirtuins. This family of enzymes is known for its role in all the reactions needing NAD+ coenzymatic activities [26,27]. Upregulation of SIRT1 mRNAs is able to protect from photodamage [28]: DNA damage not properly repaired is associated with cellular senescence, apoptosis, and carcinogenesis [29]. UV penetration of the dermal layer promotes the generation of reactive oxygen species (ROS) and collagen breakdown through matrix metalloproteinases (MMPs) remodeling [30]. SIRT1 inhibits MMPs and consequently also collagen degradation. Its expression increases after UV exposure in fibroblast, both in vivo and in vitro, suggesting a role of SIRT1 in counteracting the processes of photoaging [31,32] and carcinogenesis induced by UV [33].

It was previously described that the gene expression of several sirtuins depends on the donor age or the number of cell line passages, underlying a decrease in SIRT1 levels during aging [34]. Whereas SIRT1 is most associated with metabolic activities related to collagen remodeling, SIRT2 influences oxidative stress and telomere length, being mainly involved in cellular mechanisms associated with the cell cycle, and with aging.

Interestingly, SIRT2 expression increases during the mitotic phase, modulating the progression through the cell cycle, and interfering with malignant progression [35,36]. It was observed that in addition to SIRT1, SIRT2 activity also decays with aging [37,38].

All these aging-related changes negatively affect the skin phenotype, increasing the demand for skin rejuvenating products able to counteract skin aging mechanisms. Skin represents a barrier that is difficult to cross in the transport of active molecules, due to the presence of the stratum corneum [39]. Therefore, vehiculating drugs through the skin represents an important challenge for drug delivery. Nanodevices are recently emerging in this field due to their intrinsic features.

Nanofibers are a novel nanomaterial comprising fibers characterized by high surface area, low basis weight, high strength rate, and high content of small pore size with a minimum ratio of length to thickness of 1000:1 [40,41,42,43,44]. Nanofibers can be easily produced for different applications and with various polymers. Among the polymers, poly ε-caprolactone (PCL) is of great interest for drug release nanosystems, and is already approved by the Food and Drug Administration (FDA) for human use and medical-device fabrication [45].

Polycaprolactone (PCL) nanofibers can vehiculate drugs and other molecules with controlled release, being also composed of biocompatible polymers [46]. The ability to carry various types of molecules makes PCL nanofibers a perfect device to deliver topical treatments, such as plant-derived products for skin well-being. In particular, among the different plants used in folk medicine, myrtle (*Myrtus communis* L.), an endemic herbaceous plant present in Sardinia and throughout the Mediterranean area [8], is now emerging for its peculiar properties. Myrtle berries, leaves, and brushwood are often noted for their antimicrobial, antioxidant, and anti-inflammatory properties, and for enhancing wound healing process, both in vitro and in vivo [8,47,48,49]. Moreover, the seeds of this plant are rich in hydrolyzable tannins and ellagitannins like galloylquinic acid, monogalloylhexose, ellagic acid hexoside and ellagic acid, Oenothein B, and eugeniflorin D2 [50,51]. We previously demonstrated the capability of myrtle extracts to counteract the senescence process in adipose derived stem cells [10,52].

We therefore developed a nanodevice combining nanofibers and myrtle extracts from seeds in an attempt to obtain a controlled topical release of specific phytochemicals (NanoPCL-M). We previously described the effect of the treatment with NanoPCL-M against UV-related damage on skin cell populations exposed to UV in a dynamic model within a bioreactor, highlighting the ability of NanoPCL-M to prevent aging in epidermal keratinocytes in a 3D structure, modulating the expression of stemness genes of SSCs [53].

In the present study, we aimed to evaluate the effect of-M, pre-treatment on SSCs and fibroblasts behavior after UV exposure, focusing on extracellular matrix changes. In particular, we investigated the effect of-M on the molecular events modulating stem cell senescence, including SIRT1 and SIRT2, and HAS2 gene expression. Moreover, we evaluated Collagen I production by SSCs and fibroblasts to gain insights into the consequences of our approach at the level of the extracellular environment.

## 2. Materials and Methods

### 2.1. Extract Preparations

Myrtle extracts were prepared using the by-products of the industrial preparation of myrtle liqueur as raw material. Seeds were removed from berries, freeze-dried, and powdered to obtain a homogeneous sample. Seven grams of powder were extracted twice with 60 mL of an ethanol/water solution (70% EtOH). In both extractions, the mixtures were sonicated in an ultrasonic cleaner (VWR International, Leuven, Belgium) for 1 h at 25 °C, then centrifuged at 3000× *g* for 10 min. The organic extracts were combined and filtered with Whatman 4 filter paper, evaporated to dryness under nitrogen flow to remove ethanol, then freeze-dried to remove water.

### 2.2. Nanodevice Fabrication

Nanofibers were prepared from polycaprolactone (PCL) polymeric solution through a needleless direct-current electrospinning method. Electrospinning was performed using 10% (*w/v*) PCL (MW 40,000 Wako Chemicals GmbH, Neuss, Germany) solution, wherein PCL polymeric pellets were dissolved in chloroform:ethanol at a ratio of 9:1 (v/v) on a Multispin (Nanuntio, Prague, Czech Republic) electrospinning unit, with a wire needleless electrode. The applied voltage was in a range of 57 ± 1 kV. The temperature was 24 ± 1 °C and relative humidity was 40% ± 5% during electrospinning preparation. The distance between the wire electrode and the collects was 30 cm, and the fibers produced were deposited on a non-woven supporting textile (Spunbond, Pegas Textiles, Prague, Czech Republic), as previously described [53].

### 2.3. Nanodevice Characterization

The morphology of the electrospun nanofibers was evaluated using scanning electron microscopy (SEM) on a Vega 3 SBU Tescan instrument (Tescan, Brno-Kohoutovice, Czech Republic). A piece of PCL was coated with a layer of golden nanoparticles by a Quorum Q150R apparatus (Quorum Technologies, East Sussex, United Kingdom), wherein layer thickness was 8 nm ± 2 nm. Fiber diameters were measured from SEM images taken from five arbitrarily selected areas. The fiber diameter distribution in the scaffold was evaluated from 260 independent measurements in ImageJ software.

### 2.4. Myrtle Incorporation and Release Detection

Produced nanofiber towels were sterilized with ethylene oxide and cut, then samples were soaked with the seed extract described previously [8] to obtain weak interactions between extracts and the porous surfaces of the nanofibers. Every piece of PCL nanofiber of 6 mm diameter was soaked with 10 µL of 200 mg/mL of myrtle extract dissolved in 70% ethanol and allowed to dry under a flow hood before use.

A calibration curve was constructed by quantifying known amounts of the extracts at 280 nm wavelength with a Varian50 MPR microplate reader, according to the maximum UV spectrum absorption of main phenolic compounds in the seed extract [54]. The calibration curve at 280 nm absorbance was considered valid (R^2^ = 0,9952; data not shown).

To detect the amount of the extract released, nanofiber disks were left in 200 µL PBS in 96-multi well plates during 7 timepoints of 24 h each. After every timepoint, the nanofiber disk was removed and the absorbance was measured with the microplate reader (Varian50 MPR, Microplate reader, Palo Alto, CA, USA) to evaluate the kinetic release parameters. The results were calculated based on five independent experiments, each one performed in triplicate.

### 2.5. Cell Culturing and Experimental Design

Human SSCs and Human skin fibroblasts 1 (HFF1), obtained as previously described [12,55], were cultured in a DMEM low-glucose medium (Life Technologies, Carlsbad, CA, USA), supplemented with 10% fetal bovine serum (FBS Life Technologies, Carlsbad, CA, USA), 2 mM/L-glutamine (Euroclone, Milano, Italy), and 1% penicillin/streptomycin (Euroclone, Milano, Italy).

To perform the experiment, culturing both cell types separately, while maintaining their crosstalk, HFF1 and SSCs were cultured on the bioreactor Live flow (IVTech, 55054 Massarosa LU, Italy) in the chamber’s Live Box2 (IVTech, 55054 Massarosa LU, Italy). We seeded 20,000 HFF1 and 5000 SSCs in each chamber, which were mutually connected, and connected to the reservoir through a peristaltic pump with a flow rate of 0.1 mL/min.

Cells were cultured on a bioreactor for 3 and 7 days, and divided into 3 groups, as described in Table 1.

EC and T cells were exposed to UV light (253.7 nm) of 30 W for 2 min at 10 cm distance from the lamp to induce oxidative stress.

### 2.6. Evaluation of Cell Proliferation: BrdU Assay

The BrdU assay (#6813, Cell Signaling Technology, Euroclone, Milan, Italy) is an immunoassay for the quantification of cell proliferation, based on measurement of the incorporation of 5-bromo-2′-deoxyuridine (BrdU) during DNA synthesis. Cells were seeded at a concentration of 6000 cells/well in 96-well plates, pre-treated with NanoPCL-M for 3 and 7 days, and then exposed to UV light: EC and T cells were exposed to UV light for 2 min at 10 cm distance from the lamp to induce oxidative stress. UnC samples were not exposed to UV light and were not pre-treated with NanoPCL-M. T samples were pre-treated with NanoPCL-M before UV stress exposure. Cell viability was detected by plate reader (OD 450 nm) and is expressed in OD units compared with untreated cells UnC. Data are expressed as mean ± SD, referring to the control.

### 2.7. Gene Expression of Stemness and Cell Cycle Genes

Gene expression levels were detected by Real Time-PCR. HFF1 and SSCs were exposed to the different culturing conditions described in Table 1. Total mRNA was isolated using RNeasy Mini Kit (Qiagen, 40724 Hilden, Germany) according to the manufacturer’s protocol. The quantity and purity of RNA were measured by OD 260/280 nm using a Nanodrop (Thermo Scientific, Waltham, MA, USA). Then, 2.5 ng of RNA from each sample in triplicate was reverse-transcribed and amplified by a Luna^®^ Universal One-Step RT-qPCR Kit (New England Biolabs, 240 County Road Ipswich, MA, USA) via the Thermal Cycler (Bio-Rad, Hercules, CA, USA). The qRT-PCR analysis was performed for SIRT1, SIRT2, and HAS2. All the primers were previously described [56,57]. The resulting Ct value normalization was performed on the housekeeping HPRT1, and mRNA levels are expressed as fold of change (2^−∆∆ct^) as compared to negative controls (UnC = 1).

### 2.8. Fluorescence Imaging

Cells were cultured as described above for 3 and 7 days and then were fixed with 4% paraformaldehyde for 30 min at room temperature. At the end of the incubation time, HFF1 and SSCs were permeabilized using 0.1% Triton X-100 (Life Technologies, Grand Island, NY, USA)-PBS, and incubated with 3% bovine serum albumin (BSA)-0.1% Triton X-100 in PBS (Life Technologies, USA) for 30 min. Primary rabbit anti-Collagen I antibody (Abcam, Cambridge, United Kingdom) and mouse anti-Sirt1 (1F3) antibody were incubated overnight at 4 °C. Finally, cells were washed twice in PBS and stained with the fluorescence-conjugated goat anti-rabbit IgG secondary antibody (Life Technologies, USA) and goat anti-mouse IgG secondary antibody (Life Technologies, USA) at 37 °C for 1 h in the dark. Nuclei were labeled with 1 µg/mL 4,6-diamidino-2-phenylindole (DAPI). Fluorescence images were acquired under an Olympus BX61 Motorized Fluorescence Microscope (Olympus Corporation, Tokyo, Japan).

### 2.9. Statistical Analysis

All the experiments were performed in triplicate at the least two times (*n* = 6) and data are expressed as median ± standard deviation, assuming a statistically significant *p* value of ≤ 0.05 (*).

## 3. Results

### 3.1. Nanodevice Characterization

**Figure 1:**
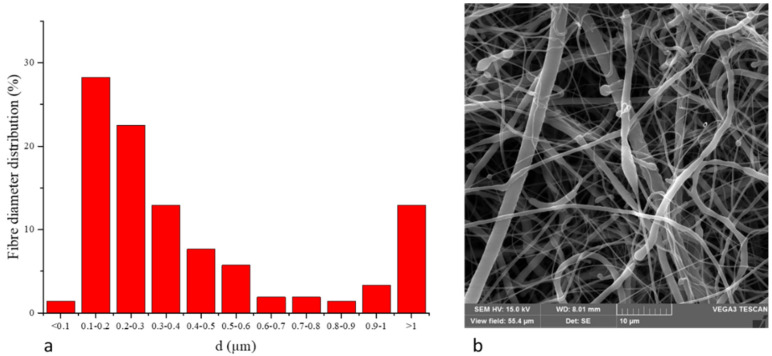
(**a**) Fiber diameter distribution of PCL nanofibers. (**b**) SEM picture of PCL nanofibers.

The PCL scaffold included polymeric fibers, wherein more than 83 % of all included fibers has diameters on a nanometric scale, called nanofibers, as illustrated in Figure 1.

### 3.2. NanoPCL-M Allows a Controlled Release of Myrtle Extracts

**Figure 2:**
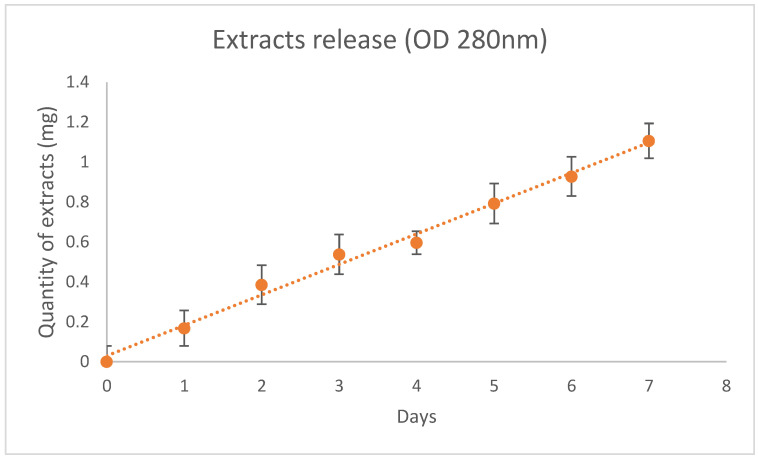
Release of myrtle extracts from NanoPCL-M for 7 days. The amount of extracts was evaluated as absorbance OD detected at 280 nm, and is expressed as mg of extracts/day.

Spectrophotometry revealed a gradual and constant release of the myrtle extracts from the PCL nanofiber device (Figure 2). The release was 0.16 ± 0.05 mg/day for 7 days.

### 3.3. NanoPCL-M Increases Cell Proliferation

**Figure 3:**
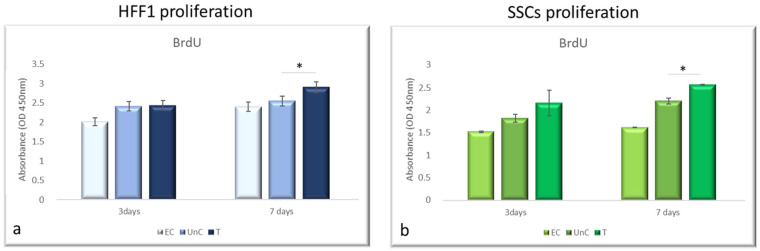
Effect of pre-treatment with NanoPCL-M on cell proliferation. HFF1 (**a**) and SSCs (**b**) were pre-treated with NanoPCL-M for 3 and 7 days, and then exposed to UV light as described. Cell viability is expressed in OD units as compared with untreated cells UnC. Data are expressed as mean ± SD referring to the control. * *p* ≤ 0.05.

The BrdU assay revealed that NanoPCL-M pre-treatment significantly increased the proliferation of the analyzed cell populations (Figure 3). The same figure shows that T samples reached a proliferation rate similar to what was observed for UnC, after 3 days of pre-treatment. Moreover, a significantly higher proliferation rate compared with UnC could be observed when cells were pre-treated for 7 days.

### 3.4. NanoPCL-M Modulates Sirtuins and Hyaluron Synthase 2 Expression under Stress Condition

**Figure 4:**
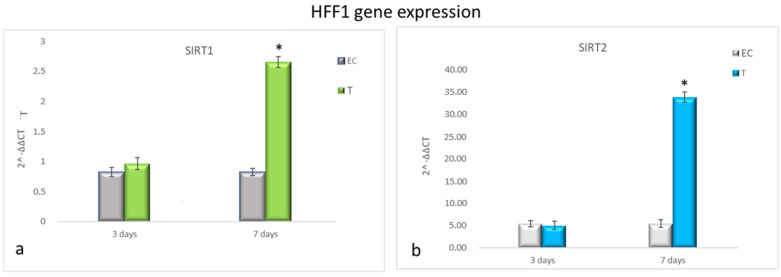
Gene expression analysis of SIRT1 and SIRT2 in HFF1 (**a**,**b**). HFF1 was exposed to the different culturing conditions. mRNA levels of SIRT1 (**a**) and SIRT2 (**b**) in EC (grey bars) and T (colored bars) are expressed as fold change (2^−∆∆ct^) compared with negative controls (UnC = 1). EC and T cells were exposed to UV light for 2 min at 10 cm distance from the lamp to induce oxidative stress. UnC samples were not exposed to UV light and not pre-treated with NanoPCL-M (UnC = 1), T samples were pre-treated with NanoPCL-M before UV stress exposure. * *p* ≤ 0.05.

**Figure 5:**
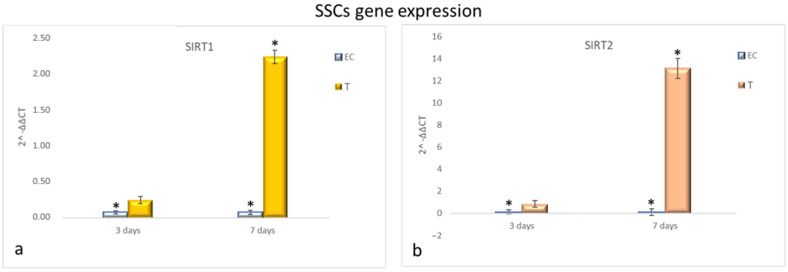
Gene expression analysis of SIRT1 and SIRT2 in SSCs (**a**,**b**). SSCs were exposed to the different culturing conditions. mRNA levels of SIRT1 (**a**) and SIRT2 (**b**) in EC (grey bars) and T (colored bars) are expressed as fold change (2^−∆∆ct^) compared with negative controls (UnC = 1). EC and T cells were exposed to UV light for 2 min at 10 cm distance from the lamp to induce oxidative stress. UnC samples were not exposed to UV light and not pre-treated with NanoPCL-M (UnC = 1). T samples were pre-treated with NanoPCL-M before UV stress exposure. * *p* ≤ 0.05.

Figure 3 shows that HFF1 pre-treated with NanoPCL-M for 3 days and then exposed to UV exhibited the same levels of SIRT1 and SIRT2 as EC (Figure 4a,b). Nevertheless, after 7 days of-M pre-treatment and UV stress, HFF1 showed a significantly higher expression of both SIRT1 and SIRT2 compared with EC (Figure 4a,b). SSCs stressed by UV (EC) showed a significant downregulation of both SIRT1 and SIRT2 compared with UnC (Figure 5a,b, grey bars). On the contrary, in Nano-PCL pre-treated SSCs (T), a significant upregulation of both SIRT1 and 2 could be observed compared with EC, starting from 3 days, and reaching a maximum after 7 days of pre-treatment, remarkably overcoming the expression level observed in unexposed controls (UnC) (Figure 5a,b).

**Figure 6:**
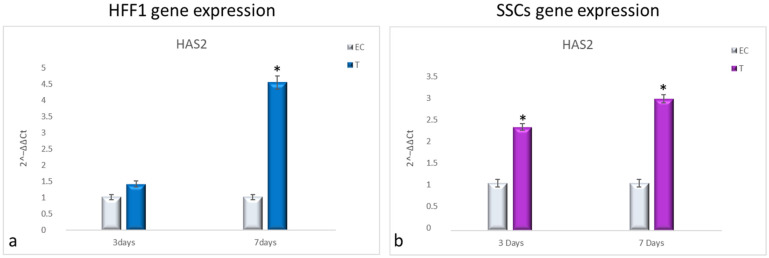
Gene expression analysis of HAS2 in HFF1 (**a**) and SSCs (**b**). HFF1 and SSCs were exposed to the different culturing conditions. mRNA levels of HAS2 in EC-HFF1 (**a**, grey bars) and T-HFF1 (blue bars). mRNA levels of HAS2 in EC-SSCs (**a**, grey bars) and T-SSCs (purple bars). mRNA levels are expressed as fold change (2^−∆∆ct^) compared with negative controls (UnC = 1). EC and T cells were exposed to UV light for 2 min at 10 cm distance from the lamp to induce oxidative stress. UnC samples were not exposed to UV light and not pre-treated with NanoPCL-M (UnC = 1). T samples were pre-treated with NanoPCL-M before UV stress exposure. * *p* ≤ 0.05.

NanoPCL-M pre-treatment induced the overexpression of HAS2 compared with EC in both HFF1 and SSCs along with all the culturing period (Figure 6a,b, respectively).

### 3.5. NanoPCL-M Preserves Collagen Depot and Modulate SIRT1 Expression under Stressing Conditions

**Figure 7:**
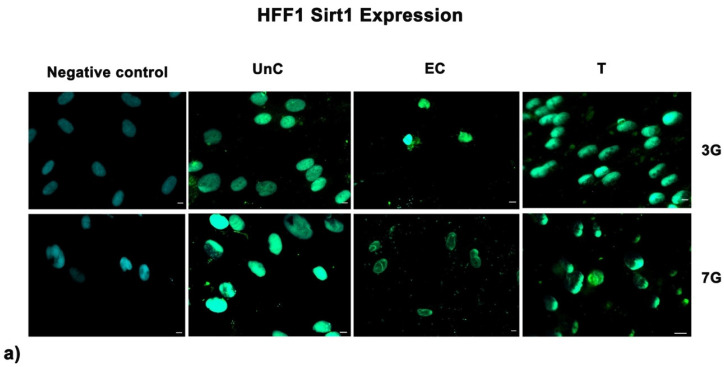

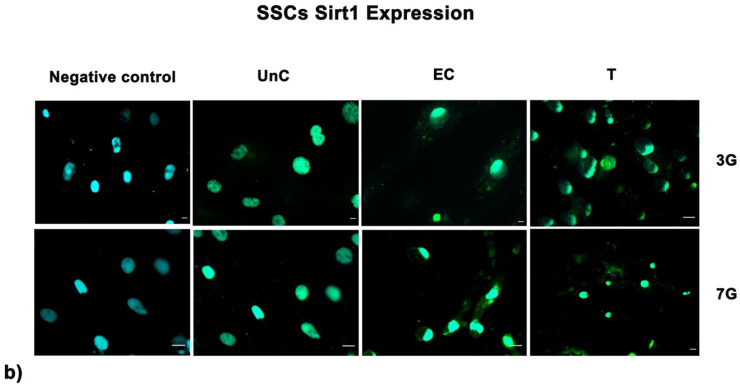
Analysis of SIRT1 expression after oxidative stress. SIRT1 images were acquired in HFF1 (**a**) and SSCs (**b**) exposed to UV treatment (EC) and cells pre-treated with NanoPCL-M and then exposed to UV radiation (T). Unexposed control cells were cultured in basic growing medium (Un-C). Cells stained with secondary antibody without any primary antibody were used as the negative control. Figures are representative of different independent experiments. For each differentiation marker, fields with the highest yield of positively stained cells are shown. Nuclei are labelled with 4,6-diamidino-2-phenylindole (DAPI, blue). Scale bars: 40 µm.

**Figure 8:**
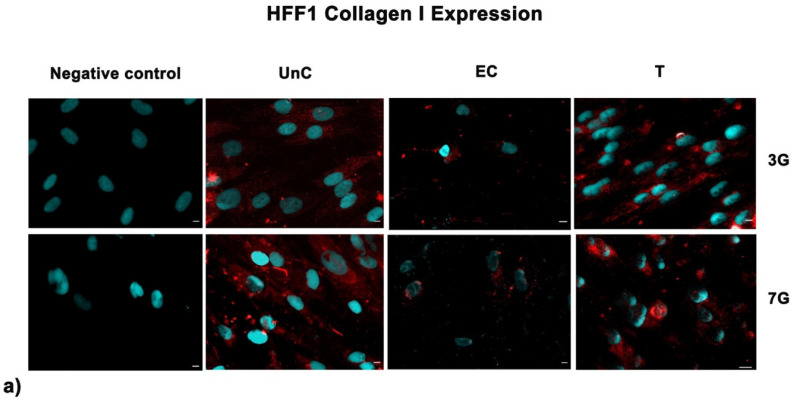

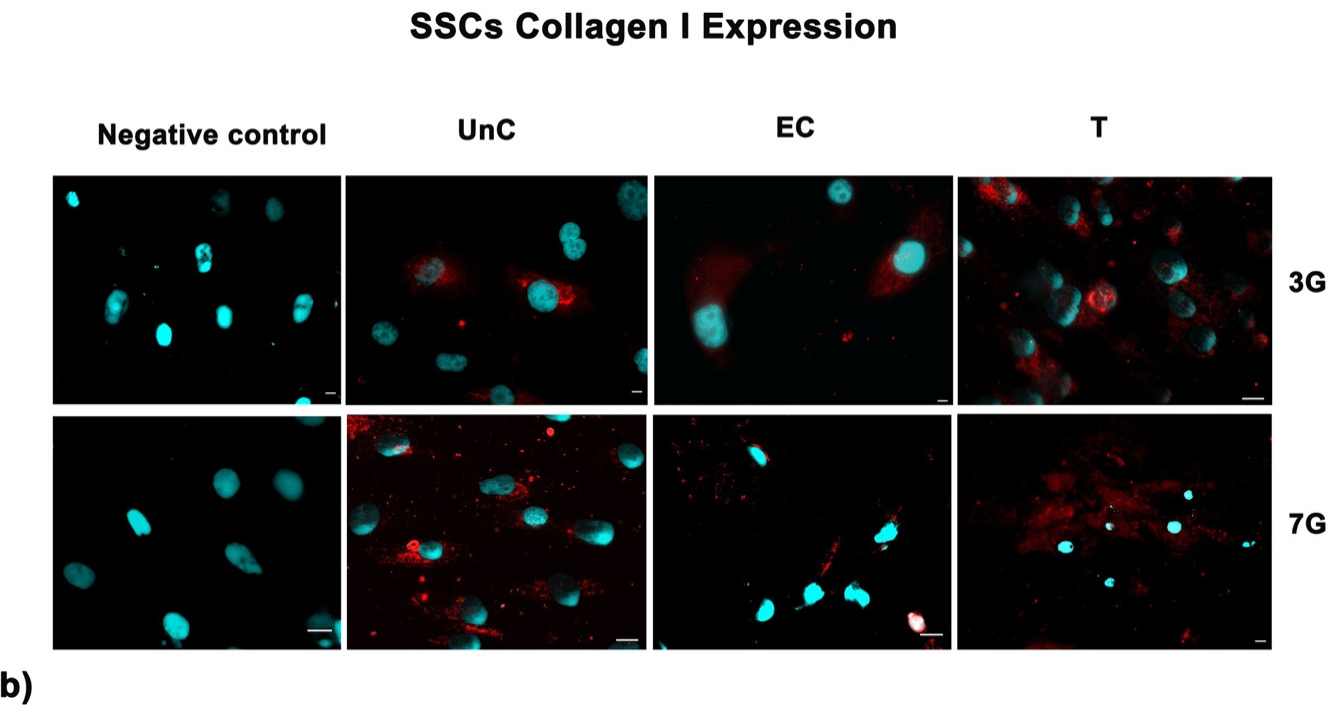
Analysis of collagen deposition after oxidative stress. Collagen type I images were acquired in HFF1 (**a**) and SSCs (**b**) exposed to UV treatment (EC) and cells pre-treated with NanoPCL-M and then exposed to UV radiation (T). Unexposed control cells were cultured in basic growing medium (UnC). Cells stained with a secondary antibody without any primary antibody were used as the negative control. Note that HFF1, or SSCs, analyzed for Collagen I expression, are the same cells that had been assessed for SIRT1 expression (Figure 6) using a double simultaneous incubation with two different antibodies, anti-mouse and anti-rabbit, as indicated in the Methods section. For each differentiation marker, fields with the highest yield of positively stained cells are shown. Nuclei are labelled with 4,6-diamidino-2-phenylindole (DAPI, blue). Scale bars: 40 µm.

Fluorescence analysis showed the higher expression of SIRT1 in cells exposed to UV-treatment (EC) and NANO-PCL (T), especially after 3 days in culture in both HFF1 (Figure 7a) and SSCs (Figure 7b) compared with untreated control cells (UnC). Moreover, pre-treatment with NanoPCL-M preserved collagen depot after oxidative stress (T) compared with unexposed controls (UnC) and exposed controls (EC) in both fibroblasts (Figure 8) and SSCs (Figure 8b).

## 4. Discussion

Stem cell behavior is deeply affected by aging that induces different cellular damages, involving changes in ECM composition and collagen remodeling [24]. The dermis, the deep layer of skin, is responsible for skin’s mechanical properties and structure, while SSCs are essential for preserving tissue homeostasis, being supported by fibroblasts for ECM maintenance [5,23]. The extracellular matrix is deeply involved in the aging process, especially in skin tissue exposed to sunlight daily [4,17].

We observed here that SSCs and HFF1 pre-treated with NanoPCL-M in vitro exhibited an upregulation of HAS2 gene expression, suggesting a possible related higher hyaluronan production. Remarkably, our results highlight that pre-treatment with NanoPCL-M is also able to preserve collagen I depot after UV-induced aging. These findings are intriguing since the HAS2 gene and collagen depot play an important role in maintaining young extracellular matrix features and skin appearance, ensuing in a proper cell cycle progression and cell turnover [26].

We previously described the anti-aging activity of NanoPCL-M on SSCs and HFF1 in reducing the amount of senescent cells and enhancing their viability [53]. In the present study, we used NanoPCL-M to obtain a controlled release of myrtle extracts in an attempt to optimize skin treatments and protect stem cells and their residing milieu. The BrdU results shown in this study reveal the ability of the nanodevice to stimulate both HFF1 and SSCs proliferation, enhancing the response to injuries and the replacement of damaged cellular elements. 

Furthermore, NanoPCL-M pre-treatment shows the ability to significantly increase the expression of the SIRT1 and SIRT2 genes. SIRT1 and SIRT2 were previously described as guardians of improper cell proliferation and may prevent carcinogenesis and melanogenesis. In this regard, it is already known that sirtuin upregulation has a main role in counteracting photoaging, as it is downregulated in aged cells [33].

Here, we reveal the capability of NanoPCL-M pre-treatment to remarkably increase the expression of SIRT1 and SIRT2 genes, strictly related to a safe proliferation and propagation in vitro of skin stem cells and HFF1, even after UV-induced aging. Considering the role of SIRT2 on telomere length regulation, this result further suggests that NanoPCL-M is able to enhance the expression of the catalytic subunit of the enzyme telomerase (TERT) in SSCs, as it was previously described by our group [53]. TERT together with the telomerase RNA component forms the most important complex involved in telomere lengthening, preserving SSC youngness [58]. PCL nanofibers, combined with Myrtle extracts, allowed for a controlled release and constant administration of bioactive molecules, perfectly addressing the need for an antiaging tool to preserve stem cells features.

## 5. Conclusions

UV radiation plays an important role in skin aging, hindering homeostasis and generating visible changes in cellular features. Here, we highlighted the protective effect of NanoPCL-M against UV exposure and its ability to increase fibroblast and skin stem cell proliferation, also influencing collagen depot and ECM organization. According to these results, we propose a potential role for our nanodevice NanoPCL-M in maintaining the features of young extracellular matrix in dermis.

## Figures and Tables

**Table 1:** The groups analyzed for both HFF1 and SSCs. EC and T cells were exposed to UV light (253.7 nm) for 2 min at 10 cm distance from the lamp to induce oxidative stress.

Name of Samples Group	Description
Unexposed control	Cells not exposed to UV light, not pre-treated with NanoPCL-M. (UnC)
Exposed control	Cells exposed to UV light and not pre-treated with NanoPCL-M. (EC)
Treated samples	Cells were exposed to UV light, after pre-treatment with NanoPCL-M (T)

## Data Availability

Not applicable.
